# Facilitators and Barriers to Participating in an Integrated Knowledge Translation Panel for a Consensus‐Building Initiative

**DOI:** 10.1111/hex.70875

**Published:** 2026-09-27

**Authors:** Simran Jawanda, Alene Toulany, Tomisin John, Dorothy Luong, Megan Agnew, Sarah E. P. Munce

**Affiliations:** ^1^ School of Medicine Queen's University Kingston Ontario Canada; ^2^ Child Health Evaluative Sciences SickKids Research Institute Toronto Ontario Canada; ^3^ Institute of Health Policy, Management and Evaluation University of Toronto Toronto Ontario Canada; ^4^ Department of Paediatrics, Division of Adolescent Medicine The Hospital for Sick Children Toronto Ontario Canada; ^5^ Department of Paediatrics, Temerty Faculty of Medicine University of Toronto Toronto Ontario Canada; ^6^ Rehabilitation Sciences Institute University of Toronto Toronto Ontario Canada; ^7^ Bloorview Research Institute Holland Bloorview Kids Rehabilitation Hospital Toronto Ontario Canada; ^8^ KITE, Toronto Rehabilitation Institute University Health Network Toronto Ontario Canada

## Abstract

**Background:**

Patient engagement is a critical component of high‐quality research. When implemented intentionally, partnerships with patient partners and other knowledge users ensure that research findings are relevant and meaningful to intended users. Integrated knowledge translation (iKT) is a collaborative approach that supports a bidirectional partnership between researchers and knowledge users throughout the research process.

**Objective:**

This study aimed to examine the experiences and identify the facilitators and barriers to participating in an iKT panel for a consensus‐building study.

**Methods:**

A qualitative descriptive study using semi‐structured interviews was conducted with members of the iKT panel, including youth, caregivers, healthcare providers, and health system leaders. The Patient Engagement in Research Scale (PEIRS‐22) was used to assess the degree of meaningful engagement. Data were analysed using a descriptive thematic approach.

**Results:**

Eleven panel members (3 youth, 2 caregivers, 2 healthcare providers, and 4 health system leaders) participated. Among the 10 participants who completed PEIRS‐22, the mean total score was 88.3 (SD = 10.5), with most reporting very or extremely meaningful (70%) level of engagement. Five overarching themes capturing the facilitators and barriers influencing panel members’ engagement were identified. Facilitators included clear and consistent communication, strong trust in and support from the research team, an inclusive and empowering environment, accessible and flexible participation options, and meaningful involvement that promoted a sense of value and connection. Key barriers included communication burden, power dynamics among panel members, competing commitments, technology challenges, and mismatched expectations.

**Conclusion:**

Engagement through an iKT approach is a complex, relational process that requires strong communication, intentional structures, and a supportive environment. Findings highlight the importance of fostering interpersonal relationships among diverse knowledge users to enhance collaboration and strengthen engagement.

**Patient and Public Contribution:**

Members of an iKT panel for a larger consensus‐building research study were participants in this evaluation. Findings will be shared with panel members to inform and refine engagement strategies for future research activities.

## 1 Introduction

There is a growing demand for patient engagement and public involvement in research to ensure research is relevant to patients’ needs [1, 2, 3, 4, 5]. Patient engagement is the active, meaningful, and collaborative involvement of individuals with lived experience to guide decision‐making in research [6]. Integrated knowledge translation (iKT) has emerged as a collaborative research approach that engages patient partners and other knowledge users throughout the research process [7, 8]. In contrast to traditional patient engagement activities, where patients’ experiences, values, and expertise are incorporated into the research, iKT emphasises an equitable, bidirectional relationship between researchers and knowledge users to co‐produce the research [7, 9]. While patient engagement and iKT approaches share similar underpinnings, the use of iKT is intended to structure the research process and create space for patient engagement. Specifically, the Canadian Institutes for Health Research (CIHR) and Canada's Strategy for Patient‐Oriented Research (SPOR) emphasise iKT as an approach to improve sustainability of research through engagement and inclusion of patient partners, physicians, system leaders and policymakers throughout the research process, consistent with engagement priorities advanced by the Patient Centred Outcomes Research Institute (PCORI) and the National Institute for Health and Care Research (NIH) [5, 10, 11, 12].

IKT emphasises authentic engagement, inclusion of knowledge users throughout the research process, and shared decision‐making [7, 13, 14]. iKT emphasises continuous engagement with patient partners, as opposed to short periods of consultation. This engagement can include collaboration on study methods, analysis, and dissemination of research findings [15]. Through the iKT process, emphasis is placed on valuing the insights and lived experience of knowledge users in co‐creating research and building knowledge [16]. Fostering a research environment centred on co‐creation can increase the uptake of research findings, making them more applicable to clinical practice, policy, and system‐level changes [10, 15].

Despite the ongoing prioritisation of patient engagement in research, significant gaps remain in the literature regarding its use, outcomes, and impact not only on the research, but also on those engaged as patient partners. Although iKT is suggested as an effective means of meaningful collaborative partnership, there have been few published evaluations to demonstrate the outcomes of the use of iKT in practice [16]. The lack of evidence on the implementation of iKT contributes to variability and uncertainty regarding how iKT should be implemented in health research [16]. For example, existing literature highlights gaps in understanding iKT terminology, relationship‐building between research teams and partners, and the impacts of iKT [2, 17]. There is a clear need for evaluations examining how knowledge users are engaged (or not) throughout the research process to identify strengths and limitations, and to minimise potential harms such as tokenistic engagement or discomfort in sharing their lived experience [16, 17, 18]. At a broader level, such efforts to evaluate iKT as a method of patient engagement align with the aims of the Guidance for Reporting Involvement of Patients and the Public (GRIPP) 2, emphasising the evaluation and reporting of patient and public involvement in research [19].

The objective of this study was to examine the experiences of knowledge users who participated as members in an integrated knowledge translation (iKT) panel within a consensus‐building study aimed at prioritising a set of quality indicator domains for transition from paediatric to adult care [20]. Specifically, we explored their perspectives on meaningful engagement in this context, including perceived facilitators and barriers.

## 2 Methods

### 2.1 Study Design

We conducted a qualitative descriptive study with iKT panel members using semi‐structured interviews. To complement the qualitative data, the Patient Engagement in Research Scale (PEIRS‐22) was administered to assess the degree of meaningful patient engagement in research [21]. This qualitative evaluation reflects the panel's experience from the initiation of our national consensus‐building study through to finalisation of a core set of quality indicators [20, 22]. A subsequent iKT evaluation is planned to examine ongoing engagement during later phases of the project, including implementation and dissemination activities.

The qualitative component of this study is reported in accordance with the Consolidated Criteria for Reporting Qualitative Research (COREQ) checklist [23]. A completed COREQ checklist is provided as Supporting File 1.

### 2.2 Study Setting and Study Context

This study was conducted at The Hospital for Sick Children (SickKids), in Toronto, Canada, as part of a national consensus‐building study to prioritise quality indicators for the transition from paediatric and adult care [20]. The broader initiative, which included an online modified Delphi (OMD) and a consensus meeting, has been reported elsewhere [22]. Briefly, the main objective was to prioritise a set of candidate quality indicator domains important and feasible to measure transition success. An iKT approach positioned knowledge users as co‐producers of research whose lived experiences informed study design, decision‐making, and outputs. At the outset, a terms of reference was co‐developed to establish a shared purpose, values and principles, roles and responsibilities, and engagement framework [13]. Within the iKT process, the iKT panel contributed to various stages of the research process (Table 1). Panel members participated in multiple meetings throughout the study, held virtually and in person, to engage in these research processes. The iKT panel was composed of youth, caregivers, healthcare providers, and health system leaders. One iKT panel member was also a youth co‐principal investigator, while all other panel members had equal roles and opportunities to participate in all engagement activities.

**Table 1:** Integration of iKT approach in the research process.

Stages	Knowledge user engagement	Output
Study design	The research team collaborated with the iKT panel members to refine the candidate quality indicator domains for the OMD study.	The candidate indicator domains reflected the perspectives of key knowledge users even before the OMD study.
Recruitment	iKT panel co‐developed recruitment strategies and contributed to the design and dissemination of recruitment materials.	Targeted recruitment approach and identification of diverse participants ensured representation of key knowledge users in transition care.
Data collection	iKT panel members informed the development of study materials that were tailored to be inclusive of youth and families’ capacity.	Appropriate and accessible plain language study materials supported an inclusive environment for all participant types.
	A subset of youth and health system leader iKT panel members participated in the Round 2 OMD discussion board moderation.	Empowered youth iKT panel members and facilitated active participation from youth and caregiver participants.
Data analyses and interpretation	Preliminary findings from the OMD study were reviewed and interpreted collaboratively with iKT panel members during a consensus meeting.	The decision on the final list of quality indicator domains reflected key knowledge users’ priorities.

### 2.3 Participants

The iKT panel consisted of four youth patient partners, three healthcare providers, five system leaders, and three caregivers. All panel members (n = 15) were invited by email to participate in this qualitative evaluation study. Although the study focused on a defined group rather than iterative sampling, a participation rate of approximately 75% was anticipated to provide sufficient thematic depth, with saturation understood as the point at which no new themes or conceptual insights emerge from the data [24].

Recruitment and interviews were conducted by a female medical student (SJ) who was not involved in the consensus‐building study. This approach was intended to minimise the potential for coercion or undue influence and to maintain separation between participants and the core research team.

### 2.4 Patient and Public Involvement

In this evaluation study, the iKT panel members participated as research participants and were invited to share their experiences of engagement using an iKT approach. The purpose and scope of the evaluation, including planned engagement methods, were discussed with panel members and documented in terms of reference [13]. This included outlining how their experiences of engagement would be evaluated.

Given that this study focused specifically on examining the experiences of iKT panel members, they were not involved in the development of the evaluation protocol, data collection materials, or data analysis. This approach was taken to minimise potential role conflict and reduce the risk of undue influence on the study findings.

### 2.5 Study Procedure

This study received approval from the Research Ethics Board at The Hospital for Sick Children (SickKids) (REB# 3473). All participants provided informed consent prior to participation.

### 2.6 Data Collection

Participants were invited to partake in semi‐structured interviews. The interview guide questions focused on participants’ experiences being part of the iKT panel and their perspectives on the engagement approaches used, including the potential barriers, facilitators and recommendations for best practices (Supporting File 2). A mock interview was conducted with a previous research team member who is also a caregiver of a young person with a chronic condition to pilot test the interview guide. The interview guide was revised based on feedback to ensure clarity and relevance. To ensure accessibility, interviews were conducted virtually using Microsoft Teams. Interviews were conducted by SJ, who had academic training and prior experience conducting qualitative interviews. Interviews were recorded, transcribed verbatim, checked for accuracy, and de‐identified to preserve participant confidentiality. Field notes were also completed after each interview. Interview transcripts were not shared with participants.

Participants were also asked to complete a sociodemographic questionnaire and the PEIRS‐22 via REDCap. The PEIRS‐22 is a validated tool used to capture data on the degree of meaningful engagement based on seven domains: procedure, convenience, contributions, team environment and interactions, support, feel valued and benefits [21]. It covers the context, process and outcome of the engagement process from the perspective of patients and caregiver research partners. Each question is rated on a 5‐point Likert scale from Strongly Agree (4), Agree (3), Neutral (2), Disagree (1) and Strongly Disagree (0). Although the PEIRS‐22 was originally designed to evaluate patient partner experience in patient engagement initiatives, the domains of the PEIRS framework capture broader features of engagement quality that are relevant to integrated knowledge translation partnerships. PEIRS‐22 has also been used in prior engagement evaluations involving mixed research teams and broader knowledge users, including community leaders, citizen partners, and clinicians [25, 26, 27]. The PEIRS framework was collaboratively selected with iKT panel members to guide the engagement approach, and so the PEIRS‐22 was chosen as a conceptually aligned tool for evaluating the quality of engagement practices and partnership between panel members and the research team. The use of PEIRS‐22 allowed us to assess the extent to which the principles informing the iKT approach were reflected in panel members’ experiences. Questionnaire data provided a structured means of quantifying these experiences and were used to complement the qualitative findings.

### 2.7 Data Analysis

Using a scoring and interpretation guide provided directly by the questionnaire creators [28], the PEIRS‐22 responses were extracted into Excel for analysis. The total score for each participant was calculated by adding the raw score across the 22 questions, dividing it by 88 (22 items X 4 scores), and then multiplying it by 100 [21, 29]. The total score across all 22 questions can range from Extreme (> 92), Very (82.7 ‐ < 92.0), Moderate (70.1 ‐ < 82.7), and Low (< 70.1) degree of meaningfulness. Interpretation of findings was based on the pre‐determined cut‐points for degree of meaningful engagement. Domain scores were the sum of each question within that section (i.e. procedural requirement included 7 items with a maximum score of 28). Each domain score can fall below or above a single pre‐determined cut‐point of low degree of meaningful engagement (Table 2).

**Table 2:** Domain‐specific cut points for low meaningfulness.

PEIRS‐22 domain	Cut point
Procedural	22.3
Convenience	9.6
Contributions	9.6
Team environment and interaction	6.4
Support	6.4
Feel values	6.4
Benefits	9.6

Interview transcripts were analysed using qualitative descriptive thematic analysis [30]. Transcripts were uploaded into NVivo version 15 (Lumivero) to support data management and coding.

Two researchers, SJ and TJ (a female research coordinator), with academic training and prior experience in qualitative methods, independently coded the transcripts. Initial open coding was conducted line by line on a subset of transcripts to remain close to participants’ language and experiences. Codes were compared and discussed iteratively, and a shared coding framework was developed through consensus. The framework was applied to the remaining transcripts and refined.

Codes were grouped into categories and synthesised into themes reflecting patterns in participants’ experiences with the iKT process. Themes were primarily organised around facilitators and barriers to engagement. Interpretive decisions and theme development were discussed with the broader analysis team to enhance analytic rigour. This team included a clinician scientist, implementation scientist and research associate with qualitative expertise, in addition to the two primary coders (AT, SM, DL, SJ, TJ). This team, except for SJ, had an established relationship with the study participants through the consensus‐building study. Preliminary findings were shared with the participants for further feedback.

Sociodemographic data were analysed descriptively to summarise participant characteristics and reported as frequencies and percentages. The PEIRS‐22 survey data were categorically analysed descriptively and used to provide contextual information regarding perceived engagement. Quantitative data were extracted from REDCap and analysed using Microsoft Excel. These data were not formally integrated (i.e., merged) with qualitative findings.

## 3 Results

### 3.1 Participant Characteristics

A total of 11 of the 15 panel members participated in the evaluation: 3 (27%) youth, 2 (18%) caregivers, 2 (18%) healthcare providers, and 4 (36%) health system leaders. Each participant was interviewed only once, with an average length of 45 min. Participant characteristics are displayed in Table 3.

**Table 3:** Participant characteristics (n = 11).

Characteristic	*n* (%)
**Gender**	
Women	9 (82)
Men	2 (18)
**Province or territory of residence**	
Ontario	9 (82)
Nova Scotia	2 (18)
**Setting of residence**	
Urban	9 (82)
Suburban/Suburb area	1 (9)
Rural	1(9)
**Current employment status**	
Employed full‐time (at 35–40 h per week)	7 (64)
Employed part‐time (up to 35 h per week)	1 (9)
Unable to work	1 (9)
Unemployed/currently looking for work	1 (9)
Self‐employed	1 (9)
**Highest level of education**	
High school	
College diploma	1 (9)
Bachelor's degree	1 (9)
Master's degree	6 (55)
PhD	1 (9)
Professional degree (MD, PsyD, PharmD, etc.)	1 (9)
Other	1 (9)
**Average household income**	
Less than $20,000	0 (0)
$20,000 to $34,999	1 (9)
$35,000 to $49,999	0 (0)
$50,000 to $74,999	2 (18)
$75,000 to $99,999	0 (0)
Over $100,000	8 (73)
**Race**	
Black	
East/Southeast Asian	1 (9)
Indigenous	1 (9)
Latino	0 (0)
Middle Eastern	0 (0)
South Asian	1 (9)
White	7 (64)
Other	1 (9)

Of the 11 participants, 10 completed the PEIRS‐22 survey: 3 youth, 2 caregivers, 2 healthcare providers, and 3 health system leaders. Total PEIRS‐22 scores revealed that 4 (40%) and 3 (30%) participants reported an extremely and very meaningful degree of engagement (Table 4). The mean total score was 88.3 (SD = 10.5), demonstrating a very meaningful degree of global meaningful engagement across all participants. Six domains were above the cut‐off point for a low degree of meaningful engagement, reflecting the positive experience voiced in the interview. The team environment and interaction domain remained at the mean cut‐off point of 6.4 (SD = 1.2). This domain included the following questions: ‘I was an equal partner in the research project team’ and ‘There was trust among the research project team members.’

**Table 4:** PEIRS‐22 scores (*N* = 10).

	PEIRS‐22 total score*	Procedural	Convenience	Contributions	Team environment and interaction	Support	Feel valued	Benefits
**Youth 3**	100	28	12	12	8	8	8	12
**Healthcare Provider 2**	98	27	12	12	8	7	8	12
**Caregiver 2**	97	28	11	11	8	8	8	11
**Youth 2**	94	28	12	11	5**	7	8	12
**Health System Leader 1**	91	27	11	10	6**	7	8	11
**Health System Leader 2**	91	27	12	11	5**	8	8	9**
**Health System Leader 3**	89	26	9**	12	6**	8	7	10
**Caregiver 1**	81	26	10	8**	6**	7	6**	8**
**Youth 1**	75	21**	9**	9**	6**	6**	6**	9**
**Healthcare Provider 1**	68**	17**	8**	8**	6**	6**	8	7**
**Mean (SD)**	88.3 (10.5)	25.5 (3.6)	10.6 (1.5)	10.4 (11.0)	6.4 (1.2)**	7.2 (0.8)	7.5 (0.8)	10.1 (1.8)
**Median**	90.9	27.0	11.0	11.0	6.0**	7.0	8.0	10.5

* Degree of meaningfulness: extremely (92.0 to 100); very (82.7 to < 92.0); moderately (70.1 to < 82.7); low (< 70.1).

** Low degree of meaningful engagement.

### 3.2 Overview of Themes

Five overarching themes and eight sub‐themes were identified, reflecting the participants’ experiences with iKT engagement. Together, these themes capture key facilitators and barriers to iKT within a consensus‐building initiative, and highlight the importance of relational, environmental, and contextual influences.

Theme 1 ***Consistency and clarity of communication from the research team***

Participants described communication as central to effective engagement. Clear, timely, and ongoing communication shaped participants’ ability to remain informed, oriented, and engaged throughout the study. Within this theme, participants described both how structured and responsive communication facilitated engagement and how communication burden and timing created challenges.

***1A. Structured and responsive communication sustains engagement***

Consistent communication was described as critical for keeping participants informed about timelines and study activities and sustaining engagement over time. Participants valued periodic email updates, regular newsletters, and clear follow‐up regarding participation and feedback opportunities.

So in that way, I think the communication was quite strong. And it's hard in these environments where you're waiting for long periods of time in‐between things, right? But, I think they did a pretty good job [of] communicating, like, enough information along the way.

– Healthcare Provider 3

Participants also highlighted strong bidirectional communication between the research team and the iKT panel, with clear points of contact, easy access to the research team, and timely responses to questions or concerns arose, all of which facilitated engagement.

I communicated the most with [research project coordinator], and they were always on top of things, always answered my emails in a timely manner, followed up on things when I wasn't sure…was very accommodating and understanding.

– Youth 2

Advanced preparation and planning for meetings further supported engagement. Receiving materials in advance and conducting well‐paced meetings helped participants feel prepared and confident in contributing at meetings. Training opportunities for completing research activities were also viewed as important, particularly given the diversity of prior research experience among iKT panel members. For example, one participant highlighted the training provided on how to conduct a scoping review.

Personalised recruitment strategies were similarly described as fostering early engagement, as individualised invitations communicated that participants’ perspectives were valued from the outset.

I think like the team was very well organised, very thoughtful in their approach. Uh, not having too many meetings, not bringing people together unnecessarily…. I think they were well thought out. Well, I mean, well planned, well‐paced.

– Health System Leader 2

***1B. Communication burden and timing can undermine engagement***

Despite largely positive communication practices, participants also described challenges related to information volume, clarity and timing. Some participants described difficulties keeping up with the volume of email communication, with youth participants in particular describing difficulties balancing this with other commitments. For some, this contributed to missed opportunities to participate in research activities.

I think for me personally, I see an e‐mail, and I'll forget about it… I think people in general, clearly myself, need to get better with responding to emails, but it's easy for things to get cluttered. Especially if they go into junk mail.

– Youth 1

Limited clarity on study components and overall research progress made it difficult for participants to decide how and when to engage with the project while balancing outside commitments. One caregiver participant suggested that a study roadmap, outlining key study components and progress could help address this challenge.

But I think actually, had I had that map, I would have known what was coming. So like if I don't know that this meeting is really going to talk about recruitment, like the invitation's always there, but when you're in the moment and you're trying to figure out what's happening in this meeting, what's going on [and] what do you need from me?

– Caregiver 1

Finally, while participants valued opportunities for feedback, several noted that earlier feedback opportunities may have further strengthened engagement.

Theme 2 ***Establishing trust and support between research team and iKT panel***

Trust between participants and the research team was identified as a critical facilitator of engagement. Participants described that early interactions, including clear communication and responsiveness from the research team, helped establish confidence in the team's reliability and intentions. Trust was further reinforced throughout the study through transparent processes and consistent follow‐up from principal investigators and research coordinators.

I think having that trusted point person, I know my point person is [research project coordinator], like knowing that is very, very important

– Caregiver 1

Participants highlighted that trust involved both feeling that their input would be taken seriously and knowing the research team was approachable and responsive. This included being able to raise questions or concerns privately, without fear of judgement, which was particularly important for youth participants.

If I had thoughts about whatever was being discussed in the meeting, I felt as though if I didn't feel comfortable speaking up in the meeting, I could e‐mail [principal investigator] or [co‐principal investigator] or [patient‐partner lead].

– Youth 2

Overall, trust developed through consistent and transparent engagement, approachable team members, and responsiveness to participant needs, forming the foundation for meaningful participation in the iKT process.

I have found it very inclusive, very collaborative, very transparent. So, it's not like they're taking information and then running away… it's a very transparent process

– Health System Leader 2

Theme 3 ***Inclusivity within the research space***

Participants described the research environment as inclusive and psychologically safe, where interpersonal connections and facilitation by the research team supported meaningful partnership. Feeling empowered, comfortable, and confident in sharing perspectives was central to participants’ experiences. Youth and caregiver participants emphasised they did not feel tokenized and that their contributions were valued. Many indicated feeling comfortable in group meetings and described enthusiasm from the research team in seeking input from individuals with lived experience. Despite largely positive experiences, participants also identified challenges related to representation, power dynamics, and task complexity, highlighting areas where inclusivity could be strengthened.

***3A. Feeling safe and empowered to contribute***

Youth and caregiver participants described feeling empowered to participate when they perceived that their lived experiences were valued and meaningfully integrated into the research process. Confidence in contributing was fostered by an environment in which participants felt their perspectives would be taken seriously, even when expressing disagreement. This sense of psychological safety supported open discussion and collaborative dialogue during meetings.

But I remember walking away being like that was a good meeting because we could have a conversation and there's other people in the room, but there's a comfort there to be able to share our perspectives openly and have disagreement.

– Health System Leader 1

***3B. Research team facilitation and inclusivity practices***

Youth and caregiver participants largely described that inclusivity in the research space was intentionally achieved through strong facilitation by the research team. Participants recalled that during meetings, the research team closely followed group discussions and actively encouraged participation from a range of diverse knowledge users. As well, while recruiting and working towards research tasks, participants described that the research team offered opportunities to participate regardless of previous research or academic background.

I loved the fact that there was such a strong focus on collaborating with patients and family and caregivers in a way that I have to say, I haven't seen that much. There's often, you know, it's not tokenistic – people have good intentions.

– Health system Leader 1

***3C. Navigating power dynamics and task complexity***

Despite supportive practices, participants reported challenges related to hierarchical differences and the complexity of research tasks.

Youth and caregivers sometimes felt hesitant to voice differing opinions in the presence of senior‐level professionals. As a result, some chose not to voice their differing views to avoid challenging professional expertise from healthcare providers and health system leaders, as they were worried about potential conflict.

I'm not really somebody who, like, if the doctor or professor or someone like that says something, I'm not going to say ‐well, this is wrong because I saw it on an article or something… So, you don't want to get to the point where you're almost clashing heads or arguing with the people that are in charge because they're not just there because of the hedge, but they also have had a lot of background or research.

– Youth 1

And I did feel like a little bit of a power imbalance and especially with me, with us, receiving [health] services. So if there is going to be a [healthcare professional] at my table, even if they're on this panel and they're wearing a dual hat or whatever, they have more power than me.

– Caregiver 1

Variability in prior research experience or task complexity also contributed to discomfort or uncertainty. These challenges were often attributed to varying levels of prior exposure to specific research tasks, which contributed to uncertainty and reduced confidence in participation.

Theme 4 ***Accessibility, flexibility, and personal factors shaping engagement***

Participants emphasised that the accessibility and flexibility of participation shaped their ability to engage on the iKT panel. Virtual options allowed for engagement regardless of geographical location or physical abilities. Participants also emphasised that this allowed for the ability to balance the scheduling of research activities with outside commitments, making participation achievable. Accommodations, such as flexible scheduling, extended deadlines, and alternative participation methods, such as providing feedback by email rather than in meetings, further facilitated engagement.

Yeah, I think that made it easier. If they're in‐person, I don't know how that would happen? Or if all the sessions, everything's happening at meetings and my schedule's busy and I can't attend most of the meetings, and I would have fell out of the loop. So, I feel like it was a good mix of this type of online communication.

– Health System Leader 1

I'm disabled so like I can't always like get out of my house and get there. And so like being able to like participate virtually and still like have that be like considered as valuable as like physically being there was really nice.

– Youth 3

At the same time, participants noted barriers to participation, including technology challenges, difficulty engaging in hybrid meetings, and conflicts with personal schedules or caregiving responsibilities. Some expressed a preference for in‐person interaction to feel connected and ensure their voices were heard.

So the barriers are still time, right? And then time to review and to properly…. I worry that you know I'm not contributing as much as I should because it's a responsibility. If you're joining something like this to commit to it, you are speaking for other people.

– Caregiver 2

Difficulty predicting future availability – “feel nervous about committing to it because I feel like I'm not going to be able to. I can't guarantee my life will align to make it all work later.

– Healthcare Provider 2

I truly think that it's more based on my availability as opposed to a specific interest in one or the other.

– Health System Leader 3

Overall, accessibility, flexibility, and personal considerations were central to enabling meaningful participation, while technological and logistical challenges could limit engagement.

Theme 5 ***Aspects of meaningful engagement ‐ feeling valued, connection, and understanding expectations***

Participants consistently described a sense of meaningful engagement in the iKT process, rooted in feelings of being valued, contributing to a larger purpose, connection to the research team and iKT group, and clear expectations of their participation. Many compared this experience to prior research participation, indicating that this project facilitated meaningful, rather than tokenistic involvement. Importantly, participants described several by‐products of this meaningful engagement, including increased confidence in research skills, greater understanding of research roles and expectations, and opportunities to develop interpersonal, leadership, and academic skills

***5A. Feeling valued and contributing to a larger purpose***

Participants emphasised that meaningful engagement was closely linked to feeling heard and included in the decision‐making process. Engagement was considered meaningful when participants perceived their contributions as impactful, whether by influencing study decisions, supporting the advancement of research, or benefiting others in the broader system. Recognition, including financial remuneration, reinforced participants’ sense of value and appreciation for their time and effort.

I felt heard and there wasn't a time that I can think of where I shared my perspective and it was ignored. I can't think of a time of that. So yeah, my memory would tell me that I was valued and heard and anything I wanted to bring up was taken into consideration.

– Youth 2

Compensation validates that kind of knowing that your voice is still continuing to be heard.

– Youth 1

Other research projects were, I've kind of just reviewed the abstract and you know, then you're a patient reviewer. But this one was a little bit more I would say of an active involvement process and like the group was very keen as well to hear from caregivers and like youth and patients.

– Caregiver 2

Participants also linked their engagement to professional or personal interest in healthcare transitions, including lived experience with the process. Engagement was intrinsically motivating when participants saw their contributions as part of a larger purpose beyond themselves.

Just the idea of being involved in something that is bigger than myself, not just within Ontario, but like across the country. And that there's people across the country that advocate, so to speak, that are trying to push for a more integrated system is very rewarding. So, that part I find is the most rewarding out of it.

– Youth 1

***5B. Connection***

Meaningful engagement was also shaped by relationships and relational opportunities, both with the research team and within the iKT panel. Participants valued connection and collaboration, but several noted a desire for more informal opportunities to interact outside of formal meetings to strengthen personal connections and group cohesion. Youth participants in particular noted the value of peer‐to‐peer connection opportunities.

I just really wanted to network. Like not necessarily in the professional way, but just to like be in a space where other people are disabled, and we're talking about it and it's like towards something that is like constructive

– Youth 3

I think one thing as a patient partner, there was a limited time to meet with the other or like even be introduced to the other patient partners. Which would have been nicer to have a separate meeting, even just us, to like connect. Would have been appreciated.

– Youth 2

Having that ability to sort of somehow come together in‐person to actually get to know who's who, and to have that stronger sense of team, I think would have been really interesting.

– Healthcare Provider 3

***5C. Clarity of expectations***

Clear communication about roles, tasks, and expectations further influenced participants’ perception of engagement. Although less frequently discussed, some participants mentioned that when expectations were mismatched or unclear, they sometimes felt peripheral or unsure of their contribution, which could reduce the sense of meaningfulness even if other aspects of engagement were positive.

So for this one, I felt very peripheral. …which was fine. That's what I was asked to do. I did, but I didn't feel like it was my research project…I was part of the supporting cast.

– Healthcare Provider 1

## 4 Discussion

This evaluation identified several key factors shaping meaningful engagement within an iKT approach. Clear consistent communication, the development of trust and support between the research team and participants, an inclusive and respectful research environment, flexible and accessible modes of participation, and a sense of value and connection were central facilitators of engagement. In contrast, barriers included communication burden, power dynamics, competing commitments, technology challenges, and mismatched expectations. In the context of limited published evaluations of iKT in practice, our findings offer practical insight into how engagement is experienced by diverse knowledge users within a consensus‐building initiative. They highlight that meaningful iKT does not occur by default, but requires intentional design, sustained relational investment, and structural supports to enable equitable and authentic participation.

The qualitative findings were supported by PEIRS‐22 results, which indicated an overall high level of meaningful engagement among participants. High scores across most domains aligned with participants’ accounts of feeling informed, supported, and valued, reinforcing the credibility of reported positive engagement experiences. Within the existing literature on iKT, interactions between the primary research team and the iKT panel are recognised as central to effective patient‐partner engagement [31].

Grounded in participatory research approaches, iKT emphasises co‐production of knowledge, mutual benefit, and shared power between researchers and knowledge users [10]. Consistent with this literature, findings from our study demonstrate that trust and sustained engagement were strongly supported by consistency and clarity of communication from the research team, beginning early in recruitment and continuing throughout the study. Importantly, our findings suggest that trust was not only established through collaboration on research activities, but also through personalised recruitment, clear and consistent points of contact, and the development of inclusive environments where iKT members felt comfortable sharing perspectives. Our findings align with previous work by Zych et al., identifying iKT initiation as a critical phase in establishing functional partnerships that support future participation [32]. Relationship building within iKT is an ongoing and iterative process that relies on both early and sustained communication, as well as the approachability and visibility of the research team.

Excessive communication and information overload emerged as barriers, particularly for participants balancing research with external commitments, aligning with previous work recognising engagement burden as a barrier [33]. We found transparency around study progress and deliverables is essential for establishing trust between knowledge users and the research team that supports sustaining engagement over time, aligning with recommendations by Nielssen et al. (2024) [34]. These findings highlight the need for balanced, well‐coordinated communication strategies that support engagement without increasing burden. Taken together, findings emphasise bidirectional communication as a foundational mechanism shaping engagement within iKT, influencing trust, clarity of expectations, and participants’ capacity to remain involved over time.

Our study emphasises the importance of interpersonal relationships among iKT panel members and the underlying motivations for engagement. Youth and family members in particular described iKT as not only a research activity, but also a space for validation and connection with others who shared similar experiences of health care transitions. These findings suggest that patient engagement may serve relational purposes alongside fulfilling research objectives, which aligns with prior research highlighting the importance of relationship‐building to support meaningful engagement and reduce perceptions of tokenism [17, 18, 35, 36]. Our findings add nuance by demonstrating how community‐building functions as an explicit motivator for engagement, particularly for youth participants.

Despite overwhelmingly positive engagement experiences, we found that power dynamics remain a critical challenge within iKT. Differences in professional background and seniority shaped participants’ comfort in voicing input, with youth and caregiver participants reporting greater hesitation in expressing perspectives that diverged from those of healthcare providers and health system leaders. While the creation of inclusive research spaces was perceived as a facilitator, findings indicate that efforts to promote inclusivity did not always fully mitigate implicit hierarchies. This tension is also reflected in the PEIRS‐22 team environment and interaction domain score, which was comparatively lower and aligned with qualitative accounts of unequal influence and residual power imbalances. These findings are consistent with prior work identifying power imbalances as a barrier to meaningful engagement [37, 38, 39]. However, our findings demonstrate that power imbalances may occur not only between researchers and patient partners, but also among iKT panel members themselves. As noted by Kothari et al., shared research spaces alone do not neutralise authority dynamics, and differential weighting of input may persist based on professional role [37]. Recommendations for explicit discussions of power, structured opportunities for input, regular check‐ins, and the co‐creation of a terms of reference document to clarify roles, expectations, and decision‐making processes are needed to build trust and mitigate tokenistic inclusion [13].

Consistent with previous research, balancing research engagement with other commitments emerged as a barrier to engagement [15]. Manageable responsibilities, flexible participation options, and accommodations supported sustained engagement across a diverse panel. Recognition of contributions, particularly through remuneration, was closely linked to participants’ sense of feeling valued, even when not a primary motivator for participation. Meaningful recognition supports sustained engagement, whereas traditional academic recognition such as co‐authorship may not hold equivalent value for all knowledge users [40].

Findings from this evaluation will be shared with iKT panel members to promote feedback and inform improvements to engagement strategies for future research activities. More broadly, our findings highlight practical considerations to support future applications of iKT and strengthen patient engagement and public involvement in health research.

### 4.1 Strengths and Limitations

Conducting the evaluation at the mid‐point of the broader consensus‐building initiative will allow findings to be shared with panellists and support iterative learning for future iKT engagement during the study. The use of an external interviewer who was not involved in the consensus‐building study further strengthened methodological rigour by reducing the risk of social desirability bias.

Several limitations should be considered when interpreting these findings. First, the evaluation was conducted within a single consensus‐building study, and findings may not be transferable to other contexts or iKT configurations. The iKT panel, while intentionally designed to include diverse representation of knowledge users, had members who were primarily white, employed full‐time, with a master's degree, and from high‐income households, living in urban areas. This may have created a unique engagement environment that limits our study's generalisability. Not all iKT panel members participated in the evaluation, and some perspectives may therefore be underrepresented (e.g., those who had a less positive experience). Secondly, the PEIRS‐22 was validated specifically to evaluate patient partner engagement in research. While its domains aligned with the engagement and partnership qualities of interest in this evaluation, its use with healthcare providers and health system leaders represents an adapted application. Some items may have been interpreted differently by these knowledge user groups as compared to patient and caregiver knowledge user groups or may not have fully captured their distinct roles and positionalities. Therefore, the PEIRS‐22 findings should be interpreted cautiously as a structured indicator of perceived engagement quality to complement the qualitative findings.

Since the evaluation was conducted at a single mid‐point time, findings reflect participants’ experiences at that stage of the research process and may not capture how engagement experiences evolved over time. Despite the use of an external interviewer who was not involved in the consensus‐building study, social desirability bias remains a possible limitation, as participants may have been inclined to report more positive experiences of engagement. However, further steps were taken to mitigate this risk, including blinding of the primary research team to individual participant responses and clear communication and preservation of participant anonymity throughout the evaluation process.

## 5 Conclusion

This evaluation provides insight into how iKT can be implemented in practice and identifies key factors shaping meaningful engagement of diverse knowledge users. Our findings highlight the central role of trust, clear and consistent communication, inclusive and supportive environments, flexible participation, and recognition of contributions in enabling engagement. At the same time, persistent challenges related to power dynamics, competing demands, and clarity of expectations highlight the complexity of operationalizing iKT in real‐world research settings. These insights contribute to the evolving evidence base on iKT and offer actionable considerations to inform future approaches to patient engagement and public involvement in health research.

## 6 Author Contributions

**Simran Jawanda:** writing – original draft, investigation, formal analysis. **Alene Toulany:** conceptualisation, supervision, writing – review and editing, methodology, validation, funding acquisition, resources. **Tomisin John:** conceptualisation, methodology, writing – original draft, writing – review and editing, formal analysis, investigation, visualisation, project administration. **Dorothy Luong:** conceptualisation, methodology, writing – review and editing, writing – original draft, visualisation, project administration, validation. **Megan Agnew:** writing – review and editing, validation. **Sarah E. P. Munce:** writing – review and editing, supervision, conceptualisation, methodology, funding acquisition, validation, resources.

## Ethics Statement

Ethics approval was obtained through the Hospital for SickKids.

## Conflicts of Interest

The authors declare no conflicts of interest.

## Supporting information


Supporting File 1

Supporting File 2

## Data Availability

The data can be obtained from the corresponding author upon request.
