# Supplementary material for: Facilitators and Barriers to Participating in an Integrated Knowledge Translation Panel for a Consensus‐Building Initiative
Source: Health Expect. 2026 Sep 27;29(5):e70875. doi: 10.1111/hex.70875 (PMC13617305; doi:10.1111/hex.70875)
Supplement: Supplementary file 2 [file HEX-29-e70875-s002.docx]

**Qualitative Interview Guide**

**Introduction:**

1. Can you tell me about why you decided to become an iKT panel member for this study?
2. Can you tell me about your involvement in the project so far?
   a. Prompt: What specific research activities were you informed about by the research team? Which research activities did you choose to participate in and why? Which research activities did you choose not to participate in and why?
   b. Prompt: What opportunities were offered to you? What roles did you have? What responsibilities did you have?
   c. Prompt: How did these experiences compare to how you’ve been involved in research projects in the past?
      i. Alternative: If this was your first time participating in a research study, how was this experience? Were your expectations of participating in a research study met?

**Goals and Expectations:**

3. What were your expectations of the iKT panel before the study began? How have they been met or not met so far?
   a. Prompt: Did you have specific goals or outcomes you were hoping to achieve by participating?
   b. Prompt: Would you have liked to be more involved throughout the research process? If so, how or in what ways?

**Engagement and Collaboration:**

4. In what ways did the research team actively seek your input or opinions?
   a. Prompt: Could anything have been done differently?
5. If you attended any of the iKT panel meetings, how did you find the experiences?
   a. Prompt: Did you feel well-prepared for the meetings?
   b. Prompt: Did you feel comfortable speaking up during these meetings?
   c. Prompt: What, if anything, was done well? What, if anything, can be improved?

**Decision-Making Process:**

6. There have been decisions made regarding the refinement of the indicators, participant recruitment and the Online Modified Delphi study. How, if at all, do you feel that your input was considered in these decisions?
   a. Prompt: Did you feel like decisions made reflected your input?

**Communication:**

7. Can you describe the communication between the research team and the iKT panel?
   a. Prompt: Were there specific methods of communication that you found particularly effective or ineffective?
   b. Prompt: Were online communication methods helpful for your participation? Did you feel engaged using online communication methods? Are there any other methods which you would have preferred?

**Facilitators and Barriers:**

8. What factors, if any, have supported your involvement and engagement as an iKT panel member so far?
9. What challenges have you encountered in the engagement process, if any?

**Satisfaction:**

10. Overall, how have you found this experience thus far?
    a. Prompt: Have you been satisfied with participation so far?
    b. Prompt: What has worked well? What hasn’t worked well?
    c. Prompt: Where can we make you feel more comfortable/engaged in the future?
    d. Prompt: Did you find that there was enough meaningful engagement for you as a panel member?

**Closing:**

11. Is there anything else you would like to share about your experience or any other feedback for the research team?
